# Transgenerational Effects of Salt Stress Imposed to Rapeseed (*Brassica napus* var. *oleifera* Del.) Plants Involve Greater Phenolic Content and Antioxidant Activity in the Edible Sprouts Obtained from Offspring Seeds

**DOI:** 10.3390/plants10050932

**Published:** 2021-05-07

**Authors:** Paolo Benincasa, Elisabetta Bravi, Ombretta Marconi, Stanley Lutts, Giacomo Tosti, Beatrice Falcinelli

**Affiliations:** 1 Department of Agricultural, Food and Environmental Sciences, University of Perugia, Borgo XX Giugno, 74, 06124 Perugia, Italy; e_bravi@yahoo.it (E.B.); ombretta.marconi@unipg.it (O.M.); giacomo.tosti@unipg.it (G.T.); beatricefalcinelli90@gmail.com (B.F.); 2 Groupe de Recherche en Physiologie végétale, Earth and Life Institute-Agronomy (ELI-A), Université catholique de Louvain, 5 (Bte 7.07.13) Place Croix du Sud, 1348 Louvain-la-Neuve, Belgium; stanley.lutts@uclouvain.be

**Keywords:** seedling, germination, salinity, phenolic acid, phytochemical, epigenetics

## Abstract

Previous research has demonstrated that rapeseed sprouts obtained under salinity demonstrate greater phenolic content and antioxidant activity compared to those sprouted with distilled water. This work aimed to test the hypothesis that these effects of salinity may persist into the next generation, so that offspring seeds of plants grown under salt stress may give edible sprouts with increased phenolic content and antioxidant activity. Plants of one rapeseed cultivar were grown in pots with 0, 100 and 200 mM NaCl, isolated from each other at flowering to prevent cross-pollination. Offspring seeds harvested from each salinity treatment were then sprouted with distilled water. We performed the extraction of free and bound phenolic fractions of sprouts and, in each fraction (methanolic extract), we determined the total polyphenols (P), flavonoids, (F), and tannins (T) with Folin–Ciocalteu reagent, the phenolic acids (PAs) by ultra-high-performance liquid chromatographs (UHPLC) analysis, and the antioxidant activity with three tests (2,2-diphenyl-1-picrylhydrazyl-hydrate, DPPH; ferric reducing antioxidant power, FRAP; 2,2′-azino-bis[3-ethylbenzothiazoline-6-sulfonic acid] diammonium salt, ABTS). Individual seed weight was slightly decreased by salinity, whereas germination performance was improved, with a lower mean germination time for salted treatments. No significant differences were observed among treatments for P, F and T, except for bound P, while, in most cases, single PAs (as free, bound and total fractions) and antioxidant activity were significantly increased in salted treatments. Our results open new perspectives for the elicitation of secondary metabolites in the offspring seeds by growing parental plants under stressing conditions, imposed on purpose or naturally occurring.

## 1. Introduction

Rapeseed (*Brassica napus* var *oleifera* Del.) is an important oilseed species worldwide, which may encounter soil salinity in some cultivation areas, in particular during crop establishment [1]. Previous research has demonstrated that salt stress tolerance of rapeseed during germination and seedling growth varies with the genotype, depending on several physiological and biochemical aspects such as mRNA synthesis, DNA replication and repair, enzyme activity, soluble sugars concentration, stomatal conductance, and mesophyll functioning (e.g., quantum yield, photochemical and non-photochemical quenching) [1,2]. All these aspects may come into play and affect germination percentage and speed, as well as seedling root and shoot growth under salinity [1,2]. Moreover, the application of salinity may involve epigenetic effects related to DNA methylation [3]. This would mean that parental plants grown under salinity may provide progeny better adapted to salt stress. In fact, transgenerational stress memory from epigenetic mechanism leading to an adaptive response to environmental stresses has been reported for *Arabidopsis thaliana* in the case of salinity, heat stress, and UV-C radiation [4,5,6,7], for *Suaeda salsa* in case of salinity [8], for rapeseed, *Triticum aestivum* (bread wheat) and *Amaranthus palmeri* in case of water deficiency [9,10,11], to say a few. It is still under debate whether the inherited response to stress is mainly due to maternal or paternal epigenetic effects, since in many cases, the parents of both gametes are subjected to the same environment. Moreover, it is still up for debate as to whether the adaptive behaviour is actually and exclusively transgenerational, because in many cases, the environmental conditions experimented by the parents persist for their whole growing cycle, thus exposing the offspring gametes and/or seeds to the parental environment, so that effects may arise from a direct induction of the stress to the offspring generation, rather than to the parents [7]. Independent of these questions, however, it is ascertained that, where a transgenerational adaptive response occurs, it is likely to be of epigenetic origin (i.e., arising from changes in gene expression), excluding relevant DNA changes, because the stress memory is achieved after just one generation and is generalized over the whole progeny population [12,13,14]. Another possibility, excluding epigenetic effects, is that the adaptive response of the progeny seeds just lays in the presence of protective compounds overproduced by the mother plant and transferred to seeds.

Regardless of the origin, the transgenerational stress memory implies that the progeny of stressed plants is somehow primed to face the potential occurrence of similar and dissimilar stresses [15], starting from the very early growth stages, which result in faster germination and greater seedling vigour. Thus, for example, Hatzig et al. [9] found that progeny seedlings from rapeseed plants grown under drought showed greater growth rate, compared to those whose parents were grown under well-watered conditions. It is of relevance that the progeny priming to tolerate stresses stands independent of the actual occurrence of the stress [14]. The transgenerational adaptive response involves metabolic mechanisms leading to changes in the contents, relative abundances and compositions of seed storage metabolites (e.g., oil and protein content, fatty acid composition, etc.), and in osmolyte accumulation and lipid peroxidation [9,11]. In rapeseed exposed to drought, the progeny exhibited also changes in the content of glucosinolates [9], indicating that the secondary metabolism may also be affected, although this was not the case for *Arabidopsis thaliana* exposed to cold environment, where glucosinolate variations after three generations were negligible [16].

Surprisingly, very few studies reported the effect of stress memory on secondary metabolites, despite these compounds, also known as phytochemicals, widely being recognized as key factors in stress tolerance, and despite the fact that they have been raising great interest in recent decades as healthy compounds in the human diet for their role as probiotics and antioxidants. A large body of literature on sprouts has focussed on the possible means to elicit their phytochemical content, as recently reviewed by Liu et al. [17] and Galieni et al. [18], from which it can be argued that applying environmental stresses during sprouting appears to be the most efficient strategy to achieve this goal. Thus, for example, Falcinelli et al. [19] found that rapeseed sprouts obtained under moderate salinity had a higher content of phenolic compounds and higher antioxidant activity. To our knowledge, no research was conceived to specifically investigate the potential elicitation of secondary metabolites by applying the stressing conditions to the parental generation, and Galieni et al. [18] first launched the idea to work on this direction.

On these bases, this work aimed to test the hypothesis that the transgenerational memory of salt stress applied to parental rapeseed plants will result in progeny sprouts with greater phenolic content and antioxidant activity.

## 2. Results

### 2.1. Seed Production and Germination, and Sprout Growth

Seed yield in mother plants was significantly and markedly decreased by salinity, ranging from 3.72 g per plant in pots watered with 0 mM NaCl (S0), to 2.42 in pots watered with 100 mM NaCl (S100), and 1.56 in pots watered with 200 mM NaCl (S200) (Table 1). The individual seed weight was also significantly decreased by salinity, although slightly and without a linear trend passing from S100 to S200 (Table 1).

For seeds incubated with distilled water, percentage germination (G) was practically the same in all treatments, but radicle emission was much faster in S200: after 24 h from the start of incubation, the radicle was visible in 84% of seeds for S200, against 46% and 28% for S100 and S0, respectively (data not shown). For this reason, the germination test was repeated applying different NaCl concentrations (0, 100, 200, 400 mM) in the germination substrate. Results of this second germination test are reported in Table 2. All treatments reached maximum percent germination (G ≥ 95%) with 0, 100 and 200 mM NaCl, whereas at 400 mM, the G was null for S0, and equal to 4% and 17% for S100 and S200, respectively. At any of the NaCl concentrations in the germination substrate, the mean germination time (MGT) was the lowest for S200 and the highest for S0.

Sprout growth parameters were not significantly affected by treatments, however there was a general trend to increase passing from S0 to S100 to S200 (Table 3).

### 2.2. Sprout Phenolic Content and Antioxidant Activity

The free, bound and total polyphenols (P), flavonoids (F) and tannins (T) were never significantly different among treatments except for bound P, although the increase in S200 compared to S0 was higher than the least significant difference for free, bound and total P and for bound T (Table 4).

On the contrary, the contents of single phenolic acids (PAs), as free, bound and total fractions, were often significantly higher in S100 and S200 compared to S0 (Table 5).

Some phenolic acids were not detectable in S0 and became considerable in S100 and S200, e.g., the free *p*-hydroxybenzoic and the bound α-resorcylic. There were also the opposite cases of free chlorogenic and ferulic acids, that were detected in S0 and not detected in the other treatments. Concerning the phenolic acids that were present in S0, the highest increases compared to S0 were observed: among free hydroxybenzoic acids, for the *p*-coumaric in S100; among free hydroxycinnamic acids, for the sinapic, salicylic and chlorogenic in S100; among bound hydroxybenzoic acids, for the *p*-coumaric in S200; among bound hydroxycinnamic acids, for the sinapic and salicylic in S200.

As far as absolute values are concerned, very high PA concentrations, as compared to the other two treatments, were recorded in S100 for free *p*-coumaric, salicylic and sinapic acids (in the order, over 3500, 3700 and 1000 µg g^−1^ of dry weight), and in S200 for bound sinapic acid (over 2000 µg g^−1^ of dry weight).

The antioxidant activity of both the free and bound fractions was significantly increased by salinity, as indicated by all the three tests. The highest increases were observed for S200 and the bound fraction (Table 6).

## 3. Discussion

Imposing salt stress to parental plants was indispensable to study whether and how the stress memory could affect the phenolic content and antioxidant activity of sprouts obtained from the progeny seeds. The 35% and 58% decreases in seed yield recorded in S100 and S200 compared to S0 demonstrate that the salt stress was effective and quite proportional to the NaCl concentration of the watering solution (Table 1). It seems noticeable that salinity levels applied to parental plants were anything but mild, considering that they persisted for the whole plant growing cycle and that salt concentration increased further in between one watering and the next, due to evapotranspiration. Similar levels may be usual for extreme halophytes like *Suaeda salsa* [8], but not for rapeseed. This confirms that the cultivar Exagone used in our experiment is very tolerant of salinity [1,20]. Plants adapted to the stressing condition by reducing markedly the number of seeds and very slightly the individual seed weight, which appears to be the best strategy to guarantee a suitable vigour to the progeny in view of the expected persistence of the stressing environment. The better germination performance of S200 seeds at any NaCl concentration in the germination substrate (Table 2) demonstrates their higher vigour, although the subsequent increase in seedling growth was not significant compared to S0 (Table 3). Faster germination and seedling growth in the offspring generation of stressed plants were also observed for rapeseed in case of water deficiency [9] and moderate sulphur deficiency [21], for *Amaranthus palmeri* in the case of low water potential (and for *Suaeda salsa* by Guo et al. [8], and for wheat in the case of water deficiency and heat stress [17].

The reasons for increased progeny vigour in salted treatments may be numerous. Our hypothesis was that the antioxidant pool might be involved, and, on this basis, we measured the content of phenolic compounds, which had been found to be increased by salinity, both in seedlings [19] and in adult plants [22].

The general lack of significant differences among treatments in the free, bound and total polyphenols, flavonoids and tannins of the progeny sprouts apparently contradicts our hypothesis, although the pairwise comparison between S200 and S0 would reveal significantly higher values for S200 in free, bound and total polyphenols and in bound tannins (Table 4).

It is to be considered that polyphenols include several kinds of compounds, which are not all necessarily involved in the response to stress and that the increase of one species might be compensated for by the decrease of another. Moreover, the plant antioxidant pool does not lay only in phenolic compounds, but also in other secondary metabolites and, finally, the extraction method may affect the species and amounts of extracted compounds [23]. In fact, the contents of most PAs (Table 5) and the antioxidant activity measured with all the three tests (Table 6) were significantly and markedly increased in sprouts of S100 and S200.

With this regard, it seems noticeable that, taking the increase in phenolic compounds and antioxidant activity as an adaptive response to the stress, this response was not always proportional to the level of stressing condition imposed: for example, no substantial variation in free and bound polyphenols was observed in S100 as compared to S0, while the increase was relevant in S200 (Table 4); similarly, the FRAP test revealed an increase of antioxidant activity that was null (in the free fraction) or moderate (in the bound fraction) for S100 and dramatic for S200 (Table 6); on the other hand, the increase in most free PAs was the highest in S100 (Table 5).

No literature is available to compare the transgenerational effects of salt stress on phenolics and antioxidant activity in rapeseed. However, it is now well established that the plant response to salt stress does not necessarily involve a linear trend and is thus not proportional to the constraint intensity: a threshold value in terms of ion accumulation within plant tissue and/or plant water deficit has been reported in several studies to trigger specific physiological and molecular responses [24]. Moreover, it is to be noticed that passing from 100 to 200 mM NaCl concentration in the solution used for watering the pots likely corresponded to more than doubling the stress. In fact, once the soil exchangeable sodium percentage is saturated, any additional input of sodium remains freely available in the soil solution, thus exerting its harmful effect towards the plant.

No comparison seems appropriate between observed data and the data reported in Falcinelli et al. [19], although both works deal with the effect of salinity on sprouts of the same rapeseed cultivar. In fact, in the previous work [19], salinity was applied during the sprouting of the F1 hybrid seeds, whereas in the present experiment, salinity was applied throughout the whole growth cycle of the parental plants (the F1 hybrid), and then the offspring F2 seeds were sprouted without salinity. Nonetheless, it is worth pointing out that the values recorded for phenolic compounds in the two experiments are in the same order of magnitude.

Results of the three antioxidant tests were substantially consistent (Table 6). Differences can stand, considering that the three tests are based on different mechanisms and thus may be sensitive to different molecules of the complex antioxidant pool present in plant tissues [25].

The variations of PAs among treatments (Table 5) are clearly linked to their role in plant stress tolerance. Salicylic acid is acting as an important plant hormone and assumes a wide range of functions in stress signalling and tolerance to salinity, especially regarding the K^+^ versus Na^+^ discrimination, regulation of Na^+^ long-distance transport, H^+^-ATPase activity involved in toxic ion compartmentation and the prevention of stress-induced K^+^ leakage [26]. Sinapic acid offers efficient potential to manage protection against salt-induced reactive oxygen species, as recently demonstrated for the mangrove species *Avicenia marina* [27]. Moreover, *p*-coumaric acid produced by phenylpropanoid pathway may be activated by Coenzyme A to produce monolignols acting as a precursor for lignin synthesis and plays a crucial role in cell wall remodelling under salt stress, allowing one to regulate cell wall extensibility and thus cell elongation, despite turgor modifications in stressed plants [28]. Chlorogenic acid has been found to increase in leaves of honeysuckle grown in saline soil as a mechanism of acclimation to salt stress [29]. Similarly, the increase of ferulic acid in rice seedlings [30] and gentisic acid in rice leaves [31] would be associated with salt tolerance. Besides their role as antioxidants in the free form phenolic acids (especially *p*-coumaric and hydroxybenzoic acid) may bind to polyamine and play a myriad of roles in stress signalling and tolerance [32].

The observed increase of phenolics and other antioxidants is of relevance not only for plant stress tolerance, but also for the nutritional value of derived plant foods. As far as sprouts are concerned, their benefits on human health are widely recognized [18,33]. With this regard, free and bound forms are known to have different fates in the human body and related different health effects. Germano et al. [34] underlined a fast absorption and a greater bioavailability for the free forms as compared to the bound ones. However, free forms may be inactivated in the stomach, while bound forms may reach the intestine, where they are metabolized by the intestinal microflora, with the release of free forms which may then exert their positive effects [34,35,36].

Since the increase in the antioxidant activity was much greater than that of PAs, is it reasonable to assume that other antioxidant species came into play. Hatzig et al. [9] observed an increase of glucosinolates in rapeseed subjected to water stress, but this does not help here, since Exagone is a “zero glucosinolates” cultivar. Further research is needed to investigate the whole metabolome.

The increase of phenolics and antioxidant activity might have epigenetic origin, however, in lack of molecular investigation, the hypothesis that phenolic acids were overproduced by stressed mother plants, transferred to the seeds and then to young seedlings during germination could not be ruled out.

## 4. Materials and Methods

### 4.1. Growth of Parental Plants and Seed Production

We used the rapeseed cultivar Exagone (Monsanto), an F1 hybrid which was found to be highly tolerant of salinity [1]. Seeds had been germinated in Petri dishes with distilled water at 0 or 100 mM NaCl and seedlings were then transferred into 20 L pots at a density of 10 seedlings per pot, then thinned after one week (when they appeared well established) to a final density of 3 plants per pot. Three different salinity levels of irrigation water were applied: 0 mM NaCl for the treatment germinated and grown in distilled water (S0); 100 and 200 mM NaCl for the treatments germinated and grown under salinity (S100, S200 respectively). A total of 4 pots per treatment were laid down outdoor, under full sunlight, in a completely randomized design. At flowering, to avoid cross-pollination among salt treatments, pots of each salt treatment were regrouped and isolated from each other with a bee-proof net until the end of flowering. Then, the net was removed, and plants were grown until seed harvest.

The pots substrate was composed of 2/3 (*w*:*w*) of potting soil and 1/3 of gross siliceous sand. The potting soil was a mix of peat, coir and green compost with the following characteristics: pH = 7.0, EC = 0.60 dS/m, porosity 90% (*v*:*v*). The initial soil nutrient composition was not known, however this appeared to not be very relevant, because the soil was periodically (from 1 to 3–4 times per week depending on the season) flushed with the NaCl solution also containing nutrients (1 g L^−1^ of Flory 9, Agrochimica SPA, Italy, with the following nutrient content in % *w*:*w*: nitrate-N 10, ammonium-N 5, P_2_O_5_ 7, K_2_O 22, MgO 6, B 0.03, Zn 0.01, Cu 0.002, Mb 0.005, Co 0.002). Repeated flushing with a surplus of watering solution was necessary to avoid progressive salt concentration. Watering was performed by hand, taking care that the solution was uniformly distributed over the top surface of the pot.

The whole growth cycle lasted about 9 months, between October and June. Outdoor air temperature was not controlled and not measured directly within the experimental location: just to give an idea, among the months interested by the rapeseed cycle, January in Perugia is the coldest month (T_mean_ = 3–5 °C) and several frosty mornings can occur throughout winter, while June is the warmest (T_mean_ = 22–25 °C). The effect of rainfall was considered as negligible, because the tops of pots were shielded to avoid any considerable rainfall water infiltration that could influence salinity levels. 

### 4.2. Sprouting of Offspring Seeds

Seeds obtained from each treatment were then used for germination trials and sprouting. The germination test was first performed in Petri dishes with 2 replicates of 50 seeds per treatment, by laying seeds over Whatman paper wetted with distilled water. Seeds were incubated in a controlled temperature chamber at 20 °C in the dark. Since treatments showed a different speed of radicle extrusion, suggesting a different seed vigour, the germination test was then repeated with NaCl solutions of 0, 100, 200 and 400 mM, with two replicates (Petri dishes) of 50 seeds per treatment.

The sprouting was performed following the methodology of Falcinelli et al. [19]. Seeds from S0, S100 and S200 were incubated on plastic trays with distilled water. Treatments were laid down according to a completely randomized block design with four replicates (trays). Each tray contained 3.6 g of seeds, corresponding to about 1000 seeds. In each tray, seeds were positioned on filter paper laid over glass balls immersed in the distilled water contained in the trays, to guarantee constant water availability while preventing anoxia. Distilled water was periodically (every two days) added to trays to restore initial tray weight, assuming that weight change was mainly due to water evaporation. The trays were kept in a growth chamber at 18 °C in the dark. After germination, the trays were placed at a light/dark regime of 16/8 h with light intensity of 200 µmol photons m^−2^s^−1^. Rapeseed sprouts were collected after the complete development of cotyledons, at 7 days after sowing (DAS). Replicates of each treatment were re-grouped two by two and stored at −20 °C until analysis.

### 4.3. Chemicals

Methanol, water, acetic acid, ethyl acetate, acetonitrile (HPLC grade), orthophosphoric acid, citric acid, sodium hydrogen phosphate, sodium hydroxide, sodium carbonate, Folin–Ciocalteu’s phenol reagent, 2,2-diphenyl-1-picrylhydrazyl-hydrate (DPPH), 2,2-azino-bis(3-ethylbenzothiazoline-6-sulfonic acid) diammonium salt (ABTS), 2,4,6tris(2-pyridyl)-s-triazine (TPTZ), gallic, α-resorcylic, tyrosol, gentisic, *p*-hydroybenzoic, 2,6-dihydroxybenzoic, *m*-hydroxybenzoic, vanillic, salicylic, syringic, homovanillic, *p*-coumaric, *m*-coumaric, *o*-coumaric, ferulic, sinapic, caffeic, and chlorogenic acid were purchased from Sigma-Aldrich (St. Louis, MO, USA). All other chemicals used were of analytical grade.

### 4.4. Preparation of Extracts and Determination of Phenolic Fractions and Antioxidant Activities

One extract was obtained from each sample and each extract was measured once. The extraction of free and bound phenolic fractions was performed as in Bravi et al. [25]. Briefly, the free fraction was obtained by adding 5 mL of a solution of methanol, water, acetic acid (70/29.5/0.5) to 1 g of frozen rapeseed; the mixture was homogenized with ultra-turrax (Janke & Kunkel & Co., IKA Labortechnik, Staufen, Germany) (1 min), ultrasonicated at room temperature (40 min) and centrifuged (10 min at 5000× *g*). The entire extraction procedure was conducted, whilst avoiding exposure to light and to high temperature, three times. The supernatants were collected, evaporated to dryness and dissolved in 1 mL of methanol before the determination of the P content and antioxidant activities. Before the phenolic acid fraction analysis, the collected extracts were dissolved in 1 mL of 30% methanol in eluent A (0.1 M citric acid and 0.2 M sodium hydrogen phosphate; 85:15; *v*/*v*). For the determination of the bound forms of phenolic acids, an alkaline hydrolysis (with 10 mL 4 M NaOH, overnight at room temperature) of the solid residues left after extraction of free phenolic forms was conducted. After the hydrolysis, the mixture, adjusted to a pH of 2, was extracted three times with 20 mL of ethyl acetate. The supernatants were collected, evaporated under a vacuum and dissolved in methanol for the determination of total polyphenol content and antioxidant activities, and in 1 mL of 30% methanol in eluent A for ultra-high-performance liquid chromatographs (UHPLC) analysis. In each fraction, we determined total polyphenols (P), flavonoids, (F), tannins (T), phenolic acids (PAs, i.e., either hydroxybenzoic acids: α-resorcylic, *p*-coumaric, *p*-hydroxybenzoic, gentisic; or hydroxycinnamic acids: chlorogenic, ferulic, salicylic, sinapic), and antioxidant activity by three assays (2,2-diphenyl-1-picrylhydrazyl-hydrate, DPPH; ferric reducing antioxidant power, FRAP; 2,2′-azino-bis[3-ethylbenzothiazoline-6-sulfonic acid] diammonium salt, ABTS), according to Benincasa et al. [37]. The P determination was performed with Folin–Ciocalteu reagent; 0.4 mL of phenolic extract was mixed with 2 mL of Folin-Ciocalteu reagent and 1.6 mL of 7.5% sodium carbonate (Na_2_CO_3_). The absorbance was read at 765 nm, after 120 min in the dark. For the determination of the F and T fractions, 1 mL of phenolic extract from free and bound fraction was used for each test. F fraction was calculated by subtracting the non-flavonoid content from P. For F determination—the extracts were mixed with hydrochloric acid 1:4 (*v*/*v*) (1 mL) and formaldehyde (0.5 mL) (Sigma-Aldrich, St. Louis, MO, USA). The mixture was kept at room temperature for 24 h. For T determination, the extract was mixed with methylcellulose (0.2 mL) (Sigma-Aldrich, St. Louis, MO, USA), ammonium sulfate (0.4 mL) (Sigma-Aldrich, St. Louis, MO, USA) and distilled water (0.4 mL). The mixture was centrifuged at 1000 *g* for 15 min. As described above for P, aliquots (0.4 mL) of extracts, either for flavonoids or tannins, were mixed with Folin–Ciocalteu reagent (1:10) and 7.5% sodium carbonate (Na_2_CO_3_, Sigma-Aldrich, St. Louis, MO, USA), and the absorbance was read at 765 nm after two hours. The F fraction was then calculated by subtracting the non-flavonoid content from P. Standard solutions of gallic acid (GA) were used to calibrate the method Gallic acid, and results were expressed as mg gallic acid equivalent (GAE) g^−1^ dry weight (DW) of sample.

In this study, ABTS, DPPH and FRAP tests were used to measure the antioxidant activity of rapeseed sprouts; the results of all tests were expressed as Trolox equivalents (TE) g^−1^ (dry basis) of sample [37]. For ABTS, an aliquot of phenolic extract was mixed with the ABTS solution, and the absorbance was read at 734 nm after 2 h in the dark. For the DPPH assay, an aliquot of phenolic extract was mixed with DPPH solution, and the absorbance was read at 515 nm after 30 min in the dark. For the FRAP assay, an aliquot of phenolic extract was mixed with the FRAP working solution and warmed at 37 °C, in the dark, for 30 min. The absorbance was read at 593 nm.

### 4.5. UHPLC Analysis of Phenolic Acids

PAs were analysed according to the method reported by Bravi et al. [25]. A SunShell C18 column (ChromaNik Technologies Inc., Osaka 552-0001 Japan, 50 mm, 2.1 mm ID) and an UHPLC system consisting of a Knauer 3950 autosampler with a 10 µL loop, a quaternary Azura P 6.1 L pump (Knauer, Berlin, Germany) coupled with an Azura MWD 2.1 L height channel UV–vis detector, were used for the separation. The separation was carried at 25 °C and at a flow rate of 0.4 mL/min. Mobile phase A consisted of 0.1 M citric acid and 0.2 M sodium hydrogen phosphate (85/15; *v*/*v*), and mobile phase B was phase A, methanol and acetonitrile (30/20/50, *v*/*v*/*v*). The pH of mobile phase A was 2.88 and the pH of mobile phase B was adjusted to 3.44 with 85% orthophosphoric acid. A chromatographic separation was achieved using the following elution gradient: mobile phase A 90% (0 min), 100% (2 min), 70% (8 min), 50% (10 min), 20% (12 min), 90% (12.5 min). The wavelengths were 254, 278, and 324 nm. The external standard method was used for the calibration and the calibration plots were constructed for standard compounds with a linearity between 0.5 and 5 µg/mL. The Clarity Chromatography Software for Windows (DataApex, Prague, Czech Republic) was used for data acquisition and elaboration. The UHPLC allowed to shorten analysis time and save solvents, while improving resolution and sensitivity as compared to conventional HPLC. The UHPLC column had an i.d. of 2.1 mm (vs. 4.6 mm of conventional HPLC) and particle size of 2 μm (vs. 3–5 μm); the flow rate was 0.4 mL/min (vs 1.0 mL/min), and the analysis time was reduced to 10 min (vs 30 min). The theoretical evaluation of peak capacity improvements by UHPLC is 4.8.

### 4.6. Statistical Analysis

All data were analyzed by one-way ANOVA according to a completely randomized design with four replicates for seed yield and individual seed weight, and two replicates for all the other data. Means were compared by Fisher’s least significant difference (LSD) at *p*-value < 0.05.

## 5. Conclusions

To date, transgenerational stress memory has been investigated exclusively for increasing plant adaptive response. This study poses a new perspective, which is the exploitation of the adaptive increase in the progeny antioxidant content for improving the nutritional quality of food. The results demonstrate that rapeseed seeds obtained from plants subjected to salt stress gave sprouts with greater phenolic content and antioxidant activity. Further research is needed to clarify whether this evidence arises from epigenetic effects or from just an overproduction of these compounds in stressed mother plants and subsequent transfer to seeds and then seedlings. The observed increase in phenolic content and antioxidant activity is of great interest, since it means that the decrease of grain yield caused by cultivation in salted soils may be partly counterbalanced by the enhanced nutritional value of grain-derived foods like sprouts. The adaptive response arises in just one generation, thus it could be intentionally induced by imposing a certain level of stress to the parental plant crop, provided that measures are adopted to avoid a progressive increase in soil salinization. On the other hand, any limiting/stressing environment, which is normally undesirable, may become of interest, especially in the case of local self-produced seeds of open-pollinating or self-pollinating species, since a reiterated stress is likely to further enhance the adaptive increase in antioxidants.

## Figures and Tables

**Table 1:** Seed yield per plant and individual seed weight in the three salinity treatments S0, S100 and S200 (i.e., 0, 100 and 200 mM of NaCl in the solution used for watering the pots throughout the whole growing cycle of the mother plants). SE is the standard error.

	Seed Yield Per Plant (g)		Individual Seed Weight (mg)	
	Mean	SE	Mean	SE
S0	3.72	0.138	3.81	0.013
S100	2.42	0.161	3.49	0.008
S200	1.56	0.215	3.59	0.015
*F test*				
*Significance*	**		**	
*LSD*	*0.557*		*0.039*	

** significant at *p* ≤ 0.01.

**Table 2:** Percentage germination (G) and mean germination time (MGT) for seeds harvested from mother plants grown with the three salinity treatments S0, S100 and S200 (i.e., 0, 100 and 200 mM of NaCl in the solution used for watering the pots throughout the whole growing cycle of the mother plants) and incubated in Petri dishes with 0, 100, 200 and 400 mM NaCl. SE is the standard error.

	Salinity Treatment for the Sprouting of the Progeny (mM NaCl)											
	0			100			200			400		
	G	MGT	SE	G	MGT	SE	G	MGT	SE	G	MGT	SE
	%	d	d	%	d	d	%	d	d	%	d	d
S0	98	1.7	0.00	100	2.0	0.01	96	2.2	0.04	0	-	-
S100	98	1.5	0.02	98	1.9	0.03	98	2.1	0.01	4	5.0	0.00
S200	99	1.2	0.00	100	1.6	0.01	95	1.9	0.04	17	3.2	0.22
*F test*												
*Significance*	*-*	**		*-*	**		*-*	*		*-*	*-*	
*LSD*	*-*	*0.054*		*-*	*0.096*		*-*	*0.157*		*-*	*-*	

** significant at *p* ≤ 0.01; * significant at *p* ≤ 0.05. The ANOVA was not performed for G since % data would have needed arcsin transformation, however differences were negligible and clearly not significant.

**Table 3:** Individual growth parameters of sprouts obtained from seeds harvested from mother plants grown under the three salinity treatments S0, S100 and S200 (i.e., 0, 100 and 200 mM of NaCl in the solution used for watering the pots throughout the whole growing cycle). SE is the standard error.

Treatment	Shoot Length		Root Length		FW Tot		DW Tot	
	mm		mm		mg		mg	
	Mean	SE	Mean	SE	Mean	SE	Mean	SE
S0	10.5	0.35	71.3	2.05	40.3	3.25	4.01	0.119
S100	11.2	0.50	79.1	4.25	43.7	1.02	4.09	0.064
S200	13.5	0.90	80.9	0.60	48.8	2.25	4.28	0.104
*F test*								
*Significance*	*n.s.*		*n.s.*		*n.s.*		*n.s.*	
*LSD*	*2.826*		*12.360*		*10.610*		*0.443*	

*n.s.* not significant.

**Table 4:** Concentration (mg of gallic acid equivalent, GAE, per g^−1^ DW) of the free, bound and total fractions of polyphenols, tannins and flavonoids in the rapeseed sprouts obtained from seeds harvested from mother plants grown under the three salinity treatments S0, S100 and S200 (i.e., 0, 100 and 200 mM of NaCl in the solution used for watering the pots throughout the whole growing cycle). Δ is the percent variation of treatments S100 and S200 as compared to S0.

Treatments	Polyphenols		Tannins		Flavonoids	
	Mean	Δ %	Mean	Δ %	Mean	Δ %
*Free*						
0–0	15.1		8.3		9.0	
100–0	16.8	11	10.0	21	10.0	10
200–0	28.0	85	16.4	98	14.3	59
*F test*						
*Significance*	*n.s.*		*n.s.*		*n.s.*	
*LSD*	*12.01*		*11.98*		*7.17*	
*Bound*						
0–0	1.6		0.3		1.1	
100–0	1.3	−20	0.6	121	0.5	−59
200–0	2.9	83	1.0	262	1.2	7
*F test*						
*Significance*	*		*n.s.*		*n.s.*	
*LSD*	*0.96*		*0.58*		*1.22*	
*Total*						
0–0	16.7		8.6		10.1	
100–0	18.1	8	10.6	24	10.4	3
200–0	30.9	85	17.4	103	15.5	53
*F test*						
*Significance*	*n.s.*		*n.s.*		*n.s.*	
*LSD*	*12.96*		*12.19*		*8.38*	

* significant at *p* ≤ 0.05; *n.s.* not significant.

**Table 5:** Concentration (µg g^−1^ DW) of the free, bound and total fractions of phenolic acids (PAs), regrouped as hydroxybenzoic and hydroxycinnamic acids, in the rapeseed sprouts obtained from seeds harvested from mother plants grown under the three salinity treatments S0, S100 and S200 (i.e., 0, 100 and 200 mM of NaCl in the solution used for watering the pots throughout the whole growing cycle). Δ is the percent variation of treatments S100 and S200 as compared to S0. N.D.: not detected; N.Q.: not quantified.

Treatment	Phenolic Acids (µg/g DW)																					
	Hydroxybenzoic Acids										Hydroxycinnamic Acids										Total PAs	
	α-Resorcylic		*p*-Coumaric		*p*-Hydroxyb.		Gentisic		∑		Chlorogenic		Ferulic		Salicylic		Sinapic		∑			
	Mean	Δ %	Mean	Δ %	Mean	Δ %	Mean	Δ %	Mean	Δ %	Mean	Δ %	Mean	Δ %	Mean	Δ %	Mean	Δ %	Mean	Δ %	Mean	Δ %
	FREE																					
0–0	648		1400		N.D.		N.D.		2048		44		55		1187		202		1488		3536	
100–0	888	37	3582	156	71	-	N.D.	-	4541	122	145	228	N.Q.	-	3728	214	1014	402	4887	228	9428	167
200–0	920	42	1793	28	117	-	N.D.	-	2831	38	44	-	N.Q.	-	1851	56	777	284	2672	80	5503	56
*F Test*																						
*Significance*	*		**		**		-		**		**		**		**		**		**		**	
*LSD*	*123*		*245*		*11*		-		*361*		*36*		*4*		*132*		*111*		*153*		*471*	
	BOUND																					
0–0	N.D.		13		N.D.		84		97		N.D.		67		136		368		571		668	
100–0	77	-	22	63	N.Q.	-	88	5	187	93	N.D.	-	80	19	219	61	522	42	820	44	1007	51
200–0	97	-	28	115	N.D.	-	38	−54	164	69	N.D.	-	129	93	424	212	2026	450	2578	352	2742	311
*F Test*																						
*Significance*	**		*n.s.*		−		**		*		−		*n.s.*		**		**		**		**	
*LSD*	*21*		*13*		−		*22*		*52*		−		*79*		*52*		*146*		*186*		*183*	
	TOTAL																					
0–0	648		1413		N.D.		84		2145		44		122		1323		570		2059		4204	
100–0	965	49	3604	155	71	-	88	5	4728	120	145	228	80	−35	3946	198	1536	169	5707	177	10435	148
200–0	1017	57	1822	29	117	-	38	−54	2994	40	44	0	129	6	2275	72	2803	391	5250	155	8245	96
*F Test*																						
*Significance*	**		**		**		**		**		**		*n.s.*		**		**		**		**	
*LSD*	*132*		*240*		*11*		*22*		*363*		*36*		*82*		*147*		*164*		*201*		*319*	

** significant at *p* ≤ 0.01; * significant at *p* ≤ 0.05; *n.s.* not significant.

**Table 6:** Antioxidant activity measured by DPPH, FRAP and ABTS tests (μmol Trolox equivalents g^−1^ DW) of the free, bound fractions of extracts in the rapeseed sprouts obtained from seeds harvested from mother plants grown under the three salinity treatments S0, S100 and S200 (i.e., 0, 100 and 200 mM of NaCl in the solution used for watering the pots throughout the whole growing cycle). Δ is the percentage variation of treatments S100 and S200 as compared to S0.

Treatment	DPPH		FRAP		ABTS	
	Mean	Δ %	Mean	Δ %	Mean	Δ %
*Free fraction*						
0–0	34.8		80.5		71.7	
100–0	63.2	81	82.7	3	105.8	48
200–0	74.8	115	188.5	134	170.9	138
*F test*						
*Significance*	**		*		**	
*LSD*	*14.044*		*88.105*		*37.508*	
*Bound fraction*						
0–0	4.4		1.0		6.3	
100–0	36.8	739	3.1	201	31.0	393
200–0	44.9	925	13.3	1200	40.7	546
*F test*						
*Significance*	**		**		**	
*LSD*	*3.761*		*4.603*		*4.882*	

** significant at *p* ≤ 0.01; * significant at *p* ≤ 0.05.

## Data Availability

The data that support the findings of this study are available on request from the corresponding author.
